# Mechanically Robust and Thermally Stable Colorful Superamphiphobic Coatings

**DOI:** 10.3389/fchem.2018.00144

**Published:** 2018-04-30

**Authors:** Ning Tian, Penglin Zhang, Junping Zhang

**Affiliations:** ^1^Key Laboratory of Clay Mineral Applied Research of Gansu Province, Lanzhou Institute of Chemical Physics, Chinese Academy of Sciences, Lanzhou, China; ^2^College of Material Science and Engineering, Lanzhou University of Technology, Lanzhou, China

**Keywords:** superoleophobic, thermal stability, palygorskite, iron oxides, wettability

## Abstract

Colorful super anti-wetting coatings are receiving growing attention, but are challenging to invent. Here, we report a general method for preparing mechanically robust and thermally stable colorful superamphiphobic coatings. A composite of palygorskite (PAL) nanorods and iron oxide red (IOR) was prepared by solid-state grinding or hydrothermal reaction, which was then modified by hydrolytic condensation of silanes to form a suspension. Superamphiphobic coatings were prepared by spray-coating the suspension onto substrates. The superamphiphobicity depends upon the surface microstructure and chemical composition, which are controllable by the PAL/IOR concentration and the solid-state grinding time. The colorful coatings show excellent superamphiphobicity with high contact angles and low sliding angles for water and various organic liquids of low surface tension, e.g., toluene and *n*-decane. The coatings also feature high mechanical, chemical and thermal stability, which is superior to all the reported colorful super anti-wetting coatings. Moreover, superamphiphobic coatings of different colors can be prepared via the same procedure using the other metal oxides instead of IOR. We believe the colorful superamphiphobic coatings may find applications in many fields like anti-climbing of oils and restoration of cultural relics, as the coatings are applicable onto various substrates.

## Introduction

Inspired by the unique water repellency of lotus leaves and legs of water striders (Neinhuis and Barthlott, 1997; Gao and Jiang, 2004), superhydrophobic coatings have received great attention. Superhydrophobic coatings have potential applications in many fields such as self-cleaning, anti-corrosion and oil/water separation, etc. (Liu et al., 2010, 2013; Bhushan and Jung, 2011; Chu et al., 2015; Li et al., 2016). However, superhydrophobic coatings can be easily wetted by liquids of low surface tension, e.g., most of organic liquids and surfactant solutions, which largely limit their applications. Different from superhydrophobic coatings, superamphiphobic coatings repel both water and liquids of low surface tension (Lu et al., 2012; Schlaich et al., 2016). However, preparation of superamphiphobic coatings is much more difficult theoretically and technically than that of the superhydrophobic ones because of much lower surface tension of organic liquids than water (Steele et al., 2008; Bellanger et al., 2014; Chu and Seeger, 2014). For example, the surface tension of water is 72.8 mN m^−1^, whereas the surface tensions of toluene and *n*-dodecane are 28.4 and 25.4 mN m^−1^, respectively. Great efforts have been made for preparing superamphiphobic coatings (Zimmermann et al., 2008; Liu et al., 2010; Cho et al., 2016; Papadopoulos et al., 2016; Qu et al., 2016). However, only a few studies reported superamphiphobic coatings from which the liquids of low surface tension (< 27.5 mN m^−1^) could roll off. For the design of superamphiphobic coatings with low sliding angles (SAs), some special micro-/nanostructures (e.g., reentrant structures, candle soots and silicone nanofilaments) (Tuteja et al., 2007; Zhang and Seeger, 2011b; Deng et al., 2012; Liu and Kim, 2014; Wong et al., 2017) and materials of very low surface energy (e.g., fluoroPOSS; Liu and Kim, 2014) have been invented. Cohen et al. designed superamphiphobic coatings by the combination of reentrant structures and fluoroPOSS (Liu and Kim, 2014), which have promoted development of superamphiphobic coatings. Zhang and Seeger invented transparent superamphiphobic coatings based on silicone nanofilaments, which showed low SA even for *n*-decane (Zhang and Seeger, 2011b). On the other hand, the mechanical stability of most of superhydrophobic and superamphiphobic coatings is low, which makes them far from practical applications (Tian et al., 2016). High mechanical stability is a hot issue in the field, especially for superamphiphobic coatings. We have prepared durable and self-healing superamphiphobic coatings repellent even to hot liquids by the combination of silanes and palygorskite (PAL) (Li and Zhang, 2016). Furthermore, the methods for preparing superamphiphobic coatings are often complicated, expensive and confined to specific substrates. A simple, cost-effective and general method remains to be explored.

For practical applications, colorful super anti-wetting coatings are receiving growing attention. Colorful superhydrophobic coatings have been prepared by using structural color (Sato et al., 2008) or coloring species like pigments and dyes (Ogihara et al., 2011; Soler et al., 2011; Li et al., 2015). Gu et al. invented colorful superhydrophobic inverse opal films by the combination of structural color and lotus effect (Gu et al., 2003). We fabricated colorful superhydrophobic coatings by modification of Maya blue-like pigments with silanes (Reinen and Lindner, 1999; Zhang et al., 2016b). Maya blue, composed of PAL and indigo, has remarkable stability to acidic and alkaline corrosion but poor thermal stability (Doménech et al., 2007; Zhang et al., 2016a). However, colorful superamphiphobic coatings are rare because it is difficult to give consideration to the color of the coatings in designing the micro-/nanostructures and surface chemical composition of superamphiphobic coatings. Dong et al. reported the first colorful superamphiphobic coatings using Maya blue-like pigments and fluorinated polysiloxane (fluoroPOS) (Dong et al., 2017). Although encouraging results have been obtained in the field of colorful super anti-wetting coatings, their thermal stability is often low owing to the fact that organic coloring species are used. For example, the colorful superamphiphobic coatings from Maya blue-like pigments and fluoroPOS faded after being kept at temperature over 150°C for 1 h (Dong et al., 2017). Replacing the organic coloring species with metal oxides should be a possible way to enhance the thermal stability. On the other hand, the mechanical stability of colorful super anti-wetting coatings remains to be improved.

Here, we report a general method for preparing mechanically robust and thermally stable colorful superamphiphobic coatings by the combination of PAL nanorods, iron oxide red (IOR, or the other iron oxides and metal oxides) and silanes. Iron oxides are environmental friendly, low-cost, and have much higher thermal stability than organic coloring species (Tian et al., 2017). First, a composite was prepared by solid-state grinding of PAL and IOR or by hydrothermal reaction of PAL with Fe(III) salts. Then, the PAL/IOR@fluoroPOS suspension was prepared by hydrolytic condensation of *1H,1H,2H,2H*-perfluorodecyltriethoxysilane (PFDTES) and tetraethoxysilane (TEOS) in the presence of the PAL/IOR composite. Finally, the PAL/IOR@fluoroPOS coatings were prepared by spray-coating the suspension onto substrates. The colorful coatings show excellent superamphiphobicity with high contact angles (CAs) and low SAs for water and various organic liquids of low surface tension. The colorful coatings also feature high mechanical, chemical and thermal stability up to 400°C.

## Experimental

### Materials

PAL was obtained from Mingguang, Anhui, China. Iron oxide red (IOR, α-Fe_2_O_3_), iron oxide yellow (IOY, FeOOH), IOO iron oxide orange (IOO, mixture of IOR and IOY), iron oxide brown (IOBR, γ-Fe_2_O_3_) and iron oxide black (IOBL, Fe_3_O_4_) were supplied by Deqing Huayuan Pigment Co. Ltd., Zhejiang, China. TEOS (99.9%) and PFDTES (97%) were bought from Gelest. Glass slides (24 mm × 50 mm) were purchased from Menzel, Braunschweig, Germany. H_2_SO_4_, anhydrous ethanol, ammonia, diiodomethane, *n*-dodecane, *n*-decane, *n*-hexadecane and toluene were purchased from China National Medicines Co., Ltd. All the reagents are analytical grade.

### Preparation of PAL/IOR@fluoroPOS suspensions

PAL (20 g) was crushed and dispersed in 200 mL of deionized water by mechanical stirring for 20 min. The PAL suspension was left to stand at room conditions for 24 h, during which quartz and other impurities in PAL settled down. Then, the PAL suspension was collected and centrifugated at 6000 rpm for 20 min. Subsequently, the purified PAL was dried in an oven at 105°C to a constant weight, and crushed for 15 s in a shredding machine. The purified PAL was filtered by passing through a 200-mesh sieve.

The PAL/IOR@fluoroPOS suspensions were prepared according to the following procedure. Typically, the mixture of PAL (1.0 g) and IOR (0.05 g) was ground manually in an agate mortar for a period of time (5, 10, 15, 20, or 30 min) to form the PAL/IOR composite. Then, 0.5 g of the PAL/IOR composite was charged into 43 mL of anhydrous ethanol in a 100 mL conical flask. The mixture was homogenized by sonication for 50 min, and then 7 mL of ammonia solution, 50 μL of TEOS and 0.6 mL of PFDTES were added under magnetic stirring. After reacting at room conditions for 8 h, the PAL/IOR@fluoroPOS suspension was formed. The suspensions with different concentration of PAL/IOR (*C*_PAL/IOR_), grinding time (*t*_grinding_), concentration of PFDTES (*C*_PFDTES_), and concentration of TEOS (*C*_TEOS_) were prepared.

### Preparation of PAL/IOR@fluoroPOS superamphiphobic coatings

The PAL/IOR@fluoroPOS coatings were prepared by spray-coating the suspensions onto the vertically placed substrates using an airbrush (INFINITY 2 in 1, Harder and Steenbeck, Germany) with 0.2 MPa N_2_. The substrates were attached onto a hot plate by double side tapes. The substrates were cleaned by washing in turn with ethanol, acetone and distilled water, and dried under N_2_ flow. The other coatings of different colors were prepared according to the same procedure by using IOY, IOO, IOBR, and IOBL instead of IOR.

### Characterization

The microstructures of the samples were observed by a field-emission scanning electron microscope (SEM, JSM-1736701F, JEOL). For SEM observation, the samples were fixed on copper stubs and coated with a layer of gold (~7 nm). The FTIR spectra of samples were recorded using a Thermo Nicolet Nexus spectropho-tometer (Thermo, Madison, USA) in the range of 4,000–400 cm^−1^ using KBr pellets. The XPS spectra were obtained using a VG Escalab 250 Xi spectrometer equipped with a monochromated Al Kα X-ray radiation source and a hemispherical electron analyzer. The XPS spectra were recorded in the constant pass energy mode with a value of 100 eV, and all binding energies were calibrated using the C 1s peak at 284.6 eV as the reference. The TGA analysis was carried out using a STA 6000 simultaneous thermal analyzer (PerkinElmer Instrument Co., Ltd. USA) in the range of 25–1,000°C at a rate of 10°C min^−1^ in N_2_ atmosphere. CAs and SAs of liquids were measured using a Contact Angle System OCA20 (Dataphysics, Germany) equipped with a tilting table at room temperature. A minimum of six readings were recorded for each sample, and the average values with standard errors were reported.

## Results and disscussion

### Preparation of PAL/IOR@fluoroPOS suspensions and coatings

**Figure 1:**
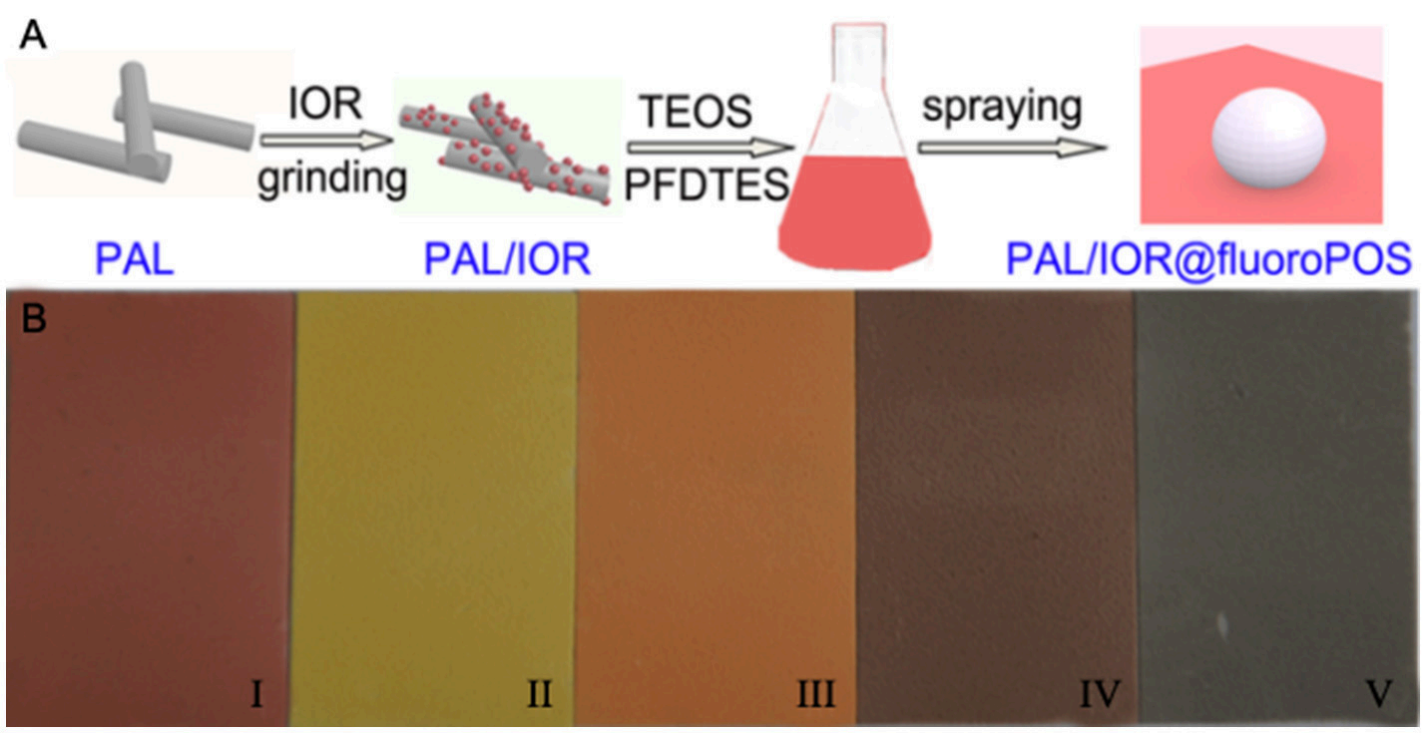
**(A)** Preparation of the PAL/IOR@fluoroPOS superamphiphobic coatings, and **(B)** images of the coatings based on (i) IOR, (ii) IOY, (iii) IOO, (iv) IOBR and (v) IOBL on glass slides. *C*_PAL/IOR_ = 14 g L^−1^, *t*_grinding_ = 20 min, *C*_PFDTES_ = 27.2 mM, *C*_TEOS_ = 4.5 mM.

Figure 1A shows the procedure for preparing the PAL/IOR@fluoroPOS superamphiphobic coatings. First, the PAL/IOR composite was prepared by solid-state grinding the mixture of PAL nanorods and IOR. Solid-state grinding could enhance the host-guest interactions, and is an important step for the fabrication of Maya blue and some functional materials (Deguchi et al., 2006; Wang et al., 2006; Koshkakaryan et al., 2009; Zhang and Li, 2013). After grinding for a period of time, the mixture of gray PAL and red IOR became a uniform red powder. Subsequently, the PAL/IOR@fluoroPOS suspensions were prepared by ammonia-catalyzed hydrolytic condensation of TEOS and PFDTES in the presence of PAL/IOR in ethanol. PFDTES tends to induce aggregation of PAL/IOR, whereas TEOS is helpful to inhibit the aggregation. The PAL/IOR@fluoroPOS superamphiphobic coatings can be readily formed by spray-coating the suspensions onto various substrates, e.g., glass slide, wood plate and aluminum foil, etc. Superamphiphobic coatings of different colors could be prepared according to the same procedure by using IOY, IOO, IOBR, and IOBL instead of IOR (Figure 1B).

**Figure 2:**
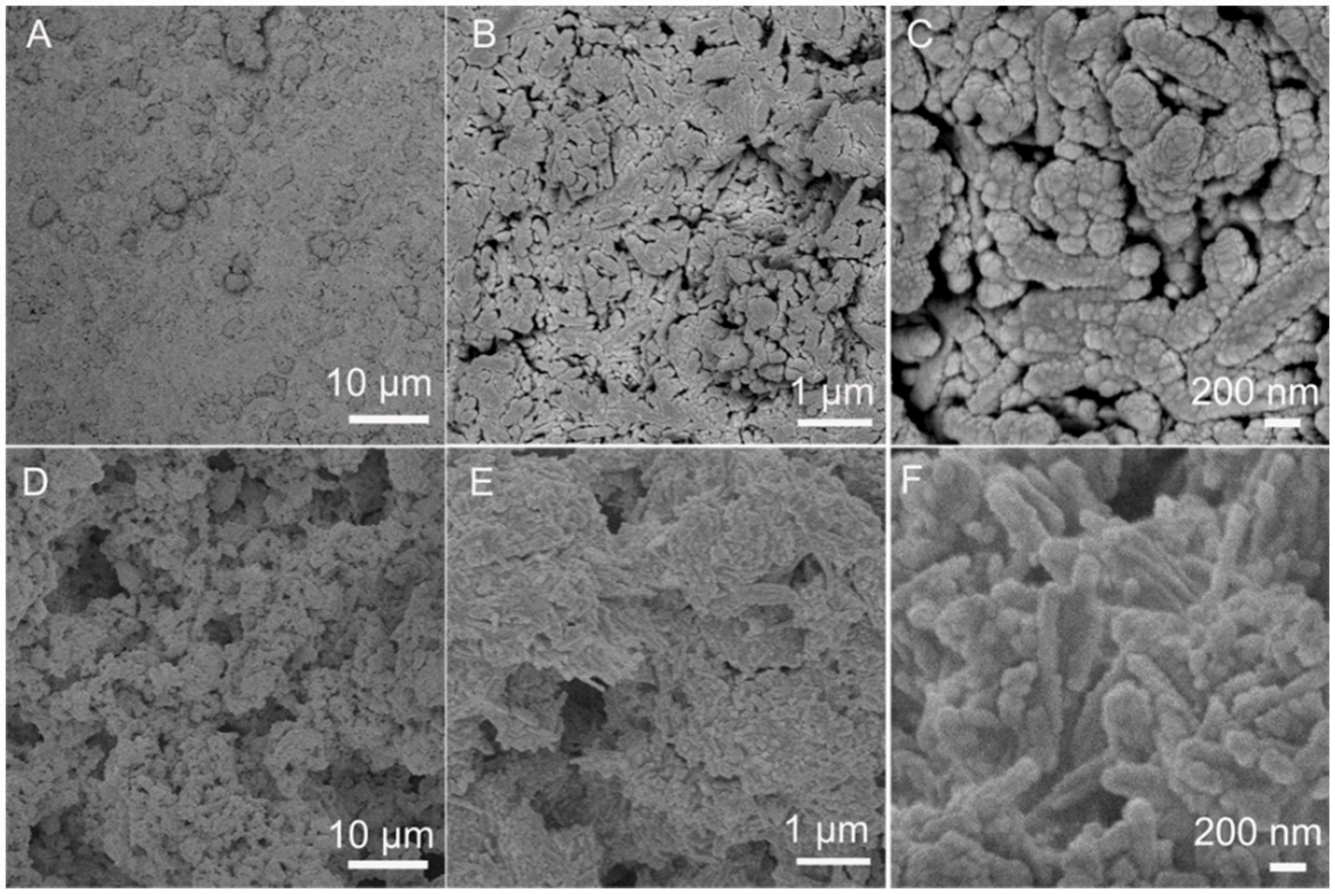
SEM images of **(A**–**C)** PAL/IOR coating and **(D**–**F)** PAL/IOR@fluoroPOS coatings. *C*_PAL/IOR_ = 14 g L^−1^, *t*_grinding_ = 20 min, *C*_PFDTES_ = 27.2 mM, *C*_TEOS_ = 4.5 mM.

The surface of the PAL/IOR coating is flat and the microscale roughness is low (Figure 2A). PAL/IOR formed nanoscale roughness of the coating owing to the nanorod-like structure of PAL (Figures 2B,C). The morphology of the PAL/IOR@fluoroPOS coating is obviously different from that of the PAL/IOR coating. The PAL/IOR@fluoroPOS coating is built up by protrusions and micropores (Figure 2D). The protrusions are composed of PAL/IOR nanorods linked together by fluoroPOS, whereas the micropores are generated by fluoroPOS-induced aggregation of PAL/IOR (Figures 2E,F). Thus, the PAL/IOR@fluoroPOS coating has a two-tier micro-/nanostructure.

**Figure 3:**
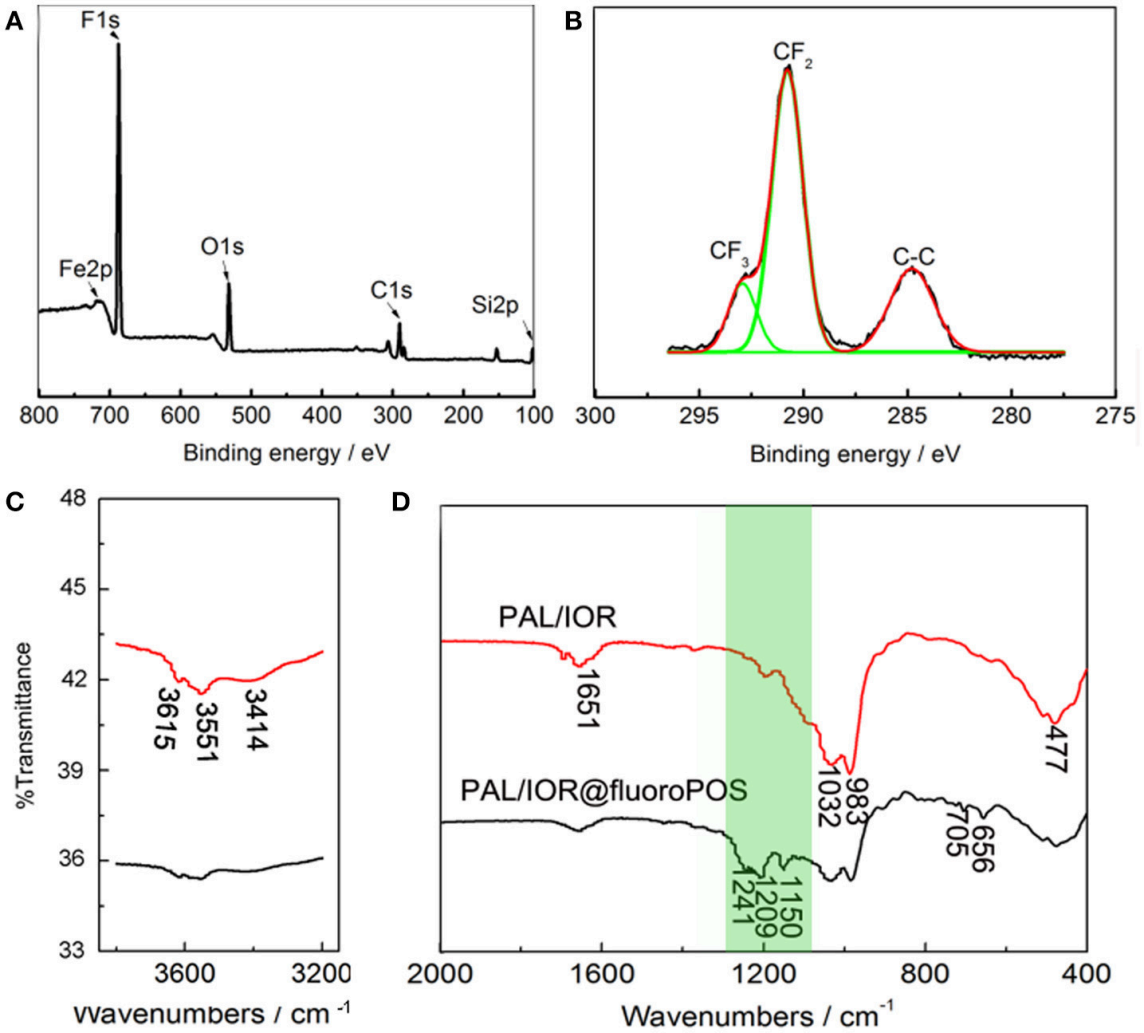
**(A)** XPS spectra, **(B)** high-resolution C 1s XPS spectrum and **(C,D)** FTIR spectra of the PAL/IOR@fluoroPOS coatings. *C*_PAL/IOR_ = 14 g L^−1^, *t*_grinding_ = 20 min, *C*_PFDTES_ = 27.2 mM, *C*_TEOS_ = 4.5 mM.

Surface chemical composition of the PAL/IOR@fluoroPOS coating was analyzed by X-ray photoelectron spectroscopy (XPS, Figures 3A,B). The peaks corresponding to C 1s (290.79 eV), O 1s (532.53 eV), Si 2p (102.61 eV), F 1s (688.46 eV), and Fe 2p (710.57 eV) were detected. The F 1s peak is very strong and the F content is as high as 46.48 at.%, which effectively decreased surface energy of the coating. The C 1s peak is attributed to the C-C, CF_2_, and CF_3_ groups. The peak corresponding to the C-O group was not detected, indicating absent of the Si-OC_2_H_5_ groups and complete hydrolysis of silanes. The chemical composition of the PAL/IOR@fluoroPOS coating was also analyzed by FTIR spectroscopy with PAL/IOR for comparison (Figures 3C,D). In the spectrum of PAL/IOR, the bands at 3,615, 3,551, and 3,414 cm^−1^ are attributed to stretching vibration of OH groups of PAL (Zhang et al., 2015), and the bands at 1,028 and 983 cm^−1^ are attributed to stretching vibration of Si-O groups of PAL. The band at 477 cm^−1^ is attributed to bending vibration of Fe-O groups of IOR and PAL. After modification with fluoroPOS, three new bands attributed to C-F groups (1,209 and 1,241 cm^−1^) and silsesquioxane (1,150 cm^−1^) were detected (Figure 3D).

### Effects of PAL/IOR on superamphiphobicity and microstructure

**Figure 4:**
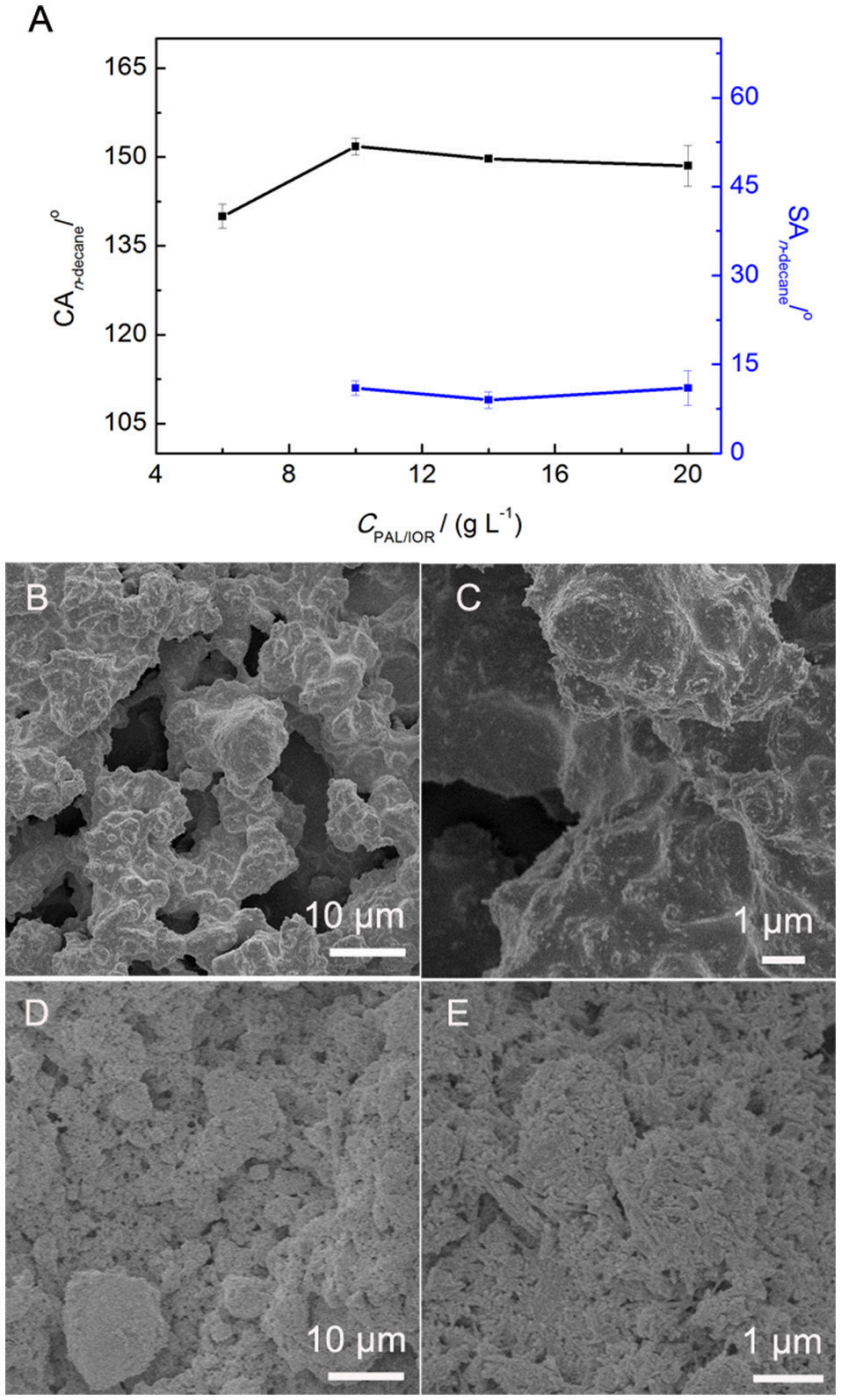
**(A)** Variation of CA_n−decane_ and SA_n−decane_ of the PAL/IOR@fluoroPOS coatings with *C*_PAL/IOR_. SEM images of the coatings with a *C*_PAL/IOR_ of **(B,C)** 6 g L^−1^ and **(D,E)** 20 g L^−1^. *t*_grinding_ = 20 min, *C*_PFDTES_ = 27.2 mM, *C*_TEOS_ = 4.5 mM.

The microstructure of the PAL/IOR@fluoroPOS coatings was constructed using the PAL/IOR composite. The PAL nanorods and IOR nanoparticles act as the skeleton of the superamphiphobic coatings, forming a two-tier hierachical micro-/nanostructure. Thus, the influences of *C*_PAL/IOR_ on superamphiphobicity and microstructure of the coatings were studied (Figure 4). Water drops have very high CA_water_ and very low SA_water_ on the surfaces of all the coatings. It is impossible to detect the difference in wettability among the coatings if water was used as the probe liquids. Thus, *n*-decane was used instead of water to show the difference in superamphiphobicity of the coatings. The *C*_PAL/IOR_ affects on the CA_n-*decane*_ and SA_n−decane_ (Figure 4A), which is owing to the change in the micro-/nanostructure of the coatings. When the *C*_PAL/IOR_ was 6 g L^−1^, the PAL/IOR nanorods were completely covered by fluoroPOS (Figures 4B,C). Thus, the coating with microscale roughness was formed, whereas the nanoscale roughness was low. Such a surface microstructure is not sufficient to make the coating superamphiphobic. The *n*-decane droplets adhered stably on the coating even when the coating was turned upside down. However, when the *C*_PAL/IOR_ increased to 10–14 g L^−1^, the superamphiphobicity was largely improved with the CA_n−decane_ about 150–152° and the SA_n−decane_ about 9–11°. The *n*-decane droplets are in the Cassie-Baxter state on the surface of the coating. For the coating with a *C*_PAL/IOR_ of 14 g L^−1^, the microscale roughness was reduced and the nanoscale was enhanced (Figures 2D,E). The protrusions and micropores distributed evenly on the surface of the coating, which could trap more air under the *n*-decane droplets. Further increasing the *C*_PAL/IOR_ to 20 g L^−1^ resulted in slight decline of the superamphiphobicity (CA_n−decane_ = 149°, SA_n−decane_ = 13°). This is attributed to the decreased surface roughness (Figures 4D,E) and the hydrophilic nature of the PAL/IOR composite.

### Effects of grinding on superamphiphobicity and microstructure

**Figure 5:**
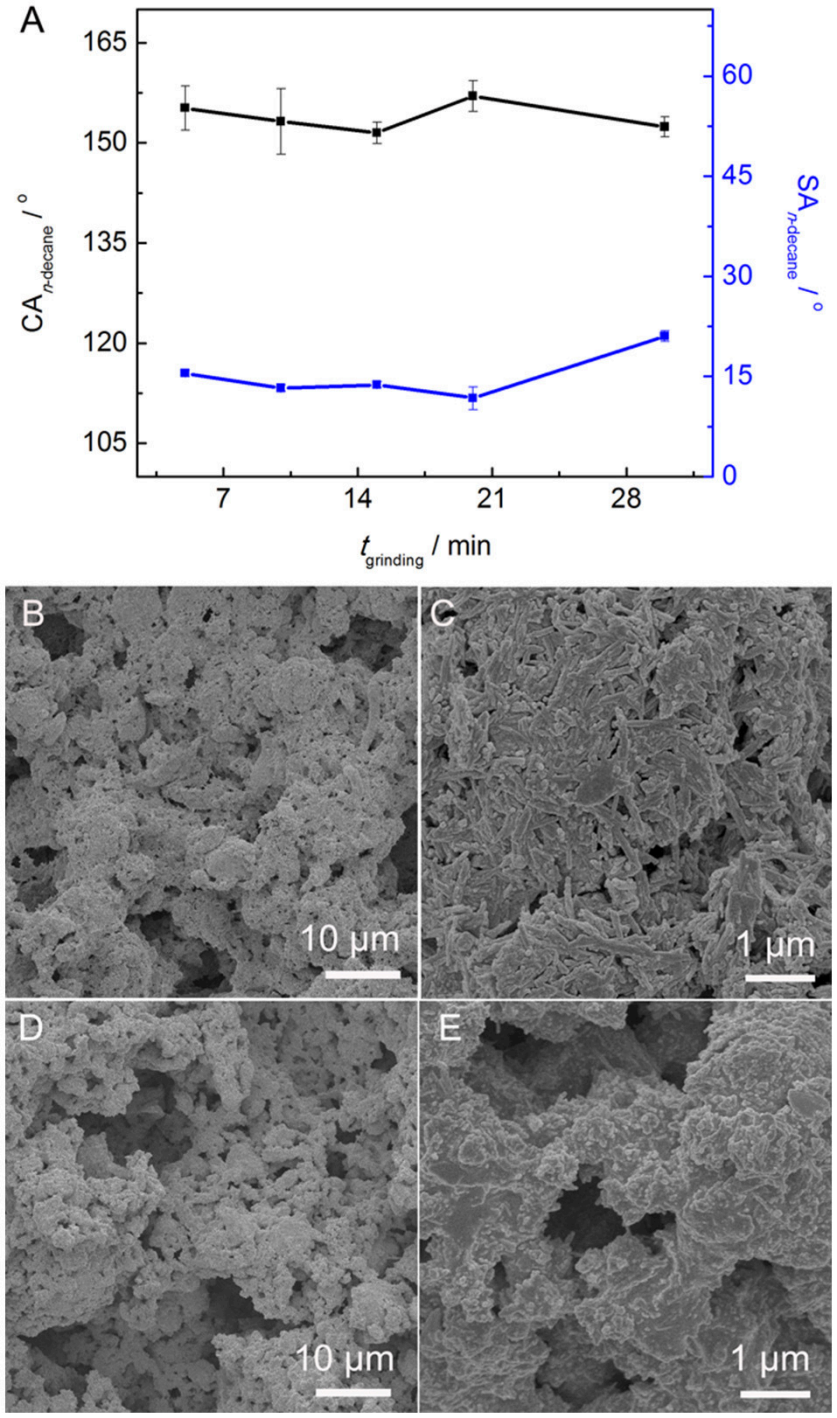
**(A)** Variation of CA_n−decane_ and SA_n−decane_ of the PAL/IOR@fluoroPOS coatings with *t*_grinding_. SEM images of the coatings with a *t*_grinding_ of **(B,C)** 5 min and **(D,E)** 30 min. *C*_PAL/IOR_ = 14 g L^−1^, *C*_PFDTES_ = 27.2 mM, *C*_TEOS_ = 4.5 mM.

Figure 5A shows the influence of *t*_grinding_ on the CA_n−decane_ and SA_n−decane_ of the PAL/IOR@fluoroPOS coatings. The CA_n−decane_ had no big change and was in the range of 155–152° with increasing the *t*_grinding_ from 5 to 30 min. Meanwhile, the SA_n−decane_ decreased from 15.5 to 12° with increasing the *t*_grinding_ to 20 min, and then increased quickly to 21° with further increasing the *t*_grinding_ to 30 min. The change in the SA_n−decane_ is consistent with the change in the surface microstructure (Figures 2D,E, 5B–E). No big difference in the surface microstructure among the coatings can be seen from the SEM images at low magnification. This means the *t*_grinding_ has no influence on microscale roughness of the coating. However, some differences among the coatings were observed from the SEM images at high magnification. The nanorods became shorter with increasing the *t*_grinding_, especially when the *t*_grinding_ was 30 min. Solid-state grinding can form the PAL/IOR composite by enhancing the interactions between PAL and IOR. Meanwhile, solid-state grinding can also dissociate the crystal bundles and aggregates of the PAL nanorods (Liu et al., 2012). This resulted in increase of nanoscale roughness and is responsible for the slight decrease of the SA_n−decane_. The PAL nanorods are about 20–30 nm in diameter and 100–1,000 nm in length, while the IOR nanoparticles are 150–300 nm in diameter (Supplementary Figure S1). However, excessive grinding resulted in shortening of the PAL nanorods (Liu et al., 2012), which are originally 500–1,000 nm in length (Supplementary Figure S2). This caused decrease of nanoscale roughness, and is responsible for the increase of the SA_n−decane_ to 21°.

### Effects of PFDTES and TEOS on superamphiphobicity and microstructure

The PAL/IOR coating is superamphiphilic, and can be easily wetted by both water and various organic liquids with CAs ≈ 0°. Here, fluoroPOS from the *in situ* hydrolytic condensation of TEOS and PFDTES was used to decrease surface energy of the coatings. The effects of *C*_PFDTES_ and *C*_TEOS_ on superamphiphobicity and microstructure of the PAL/IOR@fluoroPOS coatings were studied.

The coating with a *C*_PFDTES_ of 9.1 mM had a CA_n−decane_ of 150° and a SA_n−decane_ of 20° (Supplementary Figure S3A). With increasing the *C*_PFDTES_ to 27.2 mM, the CA_n−decane_ did not show any big change, while the SA_n−decane_ gradually decreased to 12°. This is owing to the increase of the microscale and nanoscale roughness, caused by fluoroPOS-induced aggregation of PAL/IOR (Supplementary Figures S3B–E). Also, the PAL/IOR nanorods were tightly linked together by fluoroPOS. The further increase of the *C*_PFDTES_ to 36.3 mM resulted in decline of superamphiphobicity (SA_n−decane_ = 33°). A too high *C*_PFDTES_ generated excess fluoroPOS, which buried most of the PAL/IOR nanorods and resulted in evident decrease of the surface roughness (Supplementary Figures S3F,G).

An appropriate *C*_TEOS_ could also improve the superamphiphobicity (Supplementary Figure S4A). The coating prepared without TEOS has a CA_n−decane_ of 157° and a SA_n−decane_ of 19°. With increasing the *C*_TEOS_ to 8.9 mM, the CA_n−decane_ was in the range of 157–153°, and the SA_n−decane_ gradually decreased to 11°. This is because an appropriate *C*_TEOS_ is helpful to form coatings with higher surface roughness (Supplementary Figures S4B–E). However, the further increase of the *C*_TEOS_ to 26.8 mM resulted in decrease of the CA_n−decane_ to 144° and increase of the SA_n−decane_ to 23°. This is owing to the decreased microscale roughness (Supplementary Figures S4F,G), and the hydrophilic silica formed by TEOS.

### Superamphiphobicity of PAL/IOR@fluoroPOS coatings

**Figure 6:**
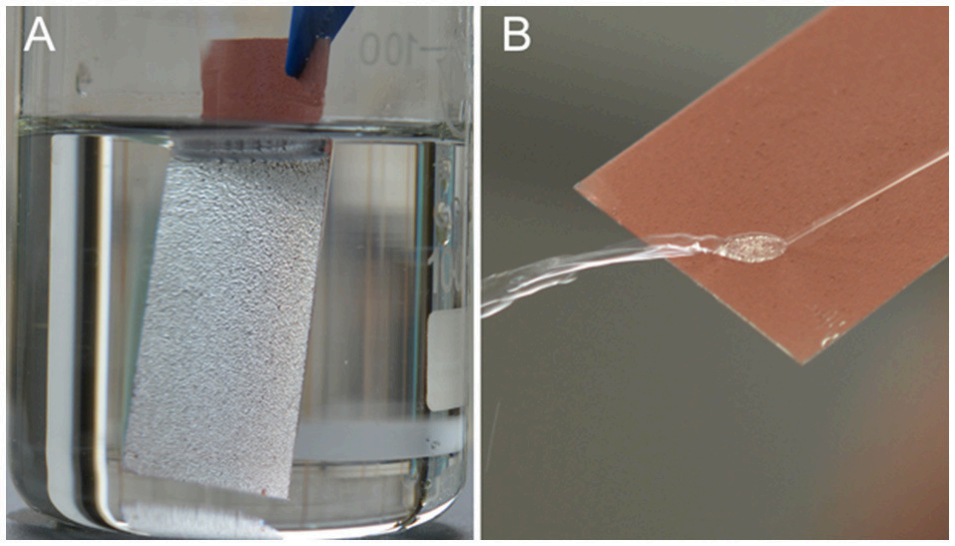
**(A)** A PAL/IOR@fluoroPOS coating immersed in *n*-hexadecane and **(B)** a jetting of *n*-hexadecane bouncing off the coating. *C*_PAL/IOR_ = 14 g L^−1^, *t*_grinding_ = 20 min, *C*_PFDTES_ = 27.2 mM, *C*_TEOS_ = 4.5 mM.

Superamphiphobicity of the PAL/IOR@fluoroPOS coating was tested by recording the CAs and SAs of many typical liquids of different surface tension (Table 1). All the liquid droplets in the table including water, toluene and *n*-decane have CAs higher than 153° and SAs lower than 13°. Also, the coating has similar CAs and SAs for many oils frequently used in our daily life including soybean oil, rapeseed oil and diesel (Supplementary Table S1). The liquid droplets are in the Cassie-Baxter state owing to the air cushion at the solid-liquid interface (Zhang and Seeger, 2011b). In addition, the liquids of lower surface tension have lower CAs and higher SAs. Furthermore, the coating showed strong light reflection once it is immersed in *n*-hexadecane (Figure 6A). A jet of *n*-hexadecane could even bounce off the coating without leaving a trace (Figure 6B). These phenomena are also attributed to the stable air cushion at the interface of the coating and *n*-hexadecane.

**Table 1:** Superamphiphobicity.

**Liquids**	**CAs/°**	**SAs/°**	**Surface tension (mN m^−1^, 20°C)**
Water	166 ± 2.1	2.0 ± 1.0	72.8
Diiodomethane	162 ± 3.5	2.3 ± 0.6	50.8
Ethylene glycol	160 ± 1.0	3.7 ± 0.6	46.49
Dichloroethane	156 ± 2.4	11.0 ± 1.7	33.3
Toluene	157 ± 0.9	10.7 ± 1.2	28.4
*n*-Hexadecane	156 ± 1.5	9.7 ± 0.6	27.5
*n*-Dodecane	154 ± 2.7	12.3 ± 1.5	25.4
*n*-Decane	153 ± 2.7	12.2 ± 1.6	23.8

*CAs and SAs of liquids (5 μL) of different surface tension on the PAL/IOR@fluoroPOS coating at 20°C. C_PAL/IOR_ = 14 g L^−1^, t_grinding_ = 20 min, C_PFDTES_ = 27.2 mM, C_TEOS_ = 4.5 mM*.

### Mechanical and chemical stability

Besides the high superamphiphobicity, the PAL/IOR@fluoroPOS coating also shows excellent mechanical and chemical stability according to the results of various stability tests, e.g., water jetting, immersion in corrosive liquids and UV irradiation, etc.

**Figure 7:**
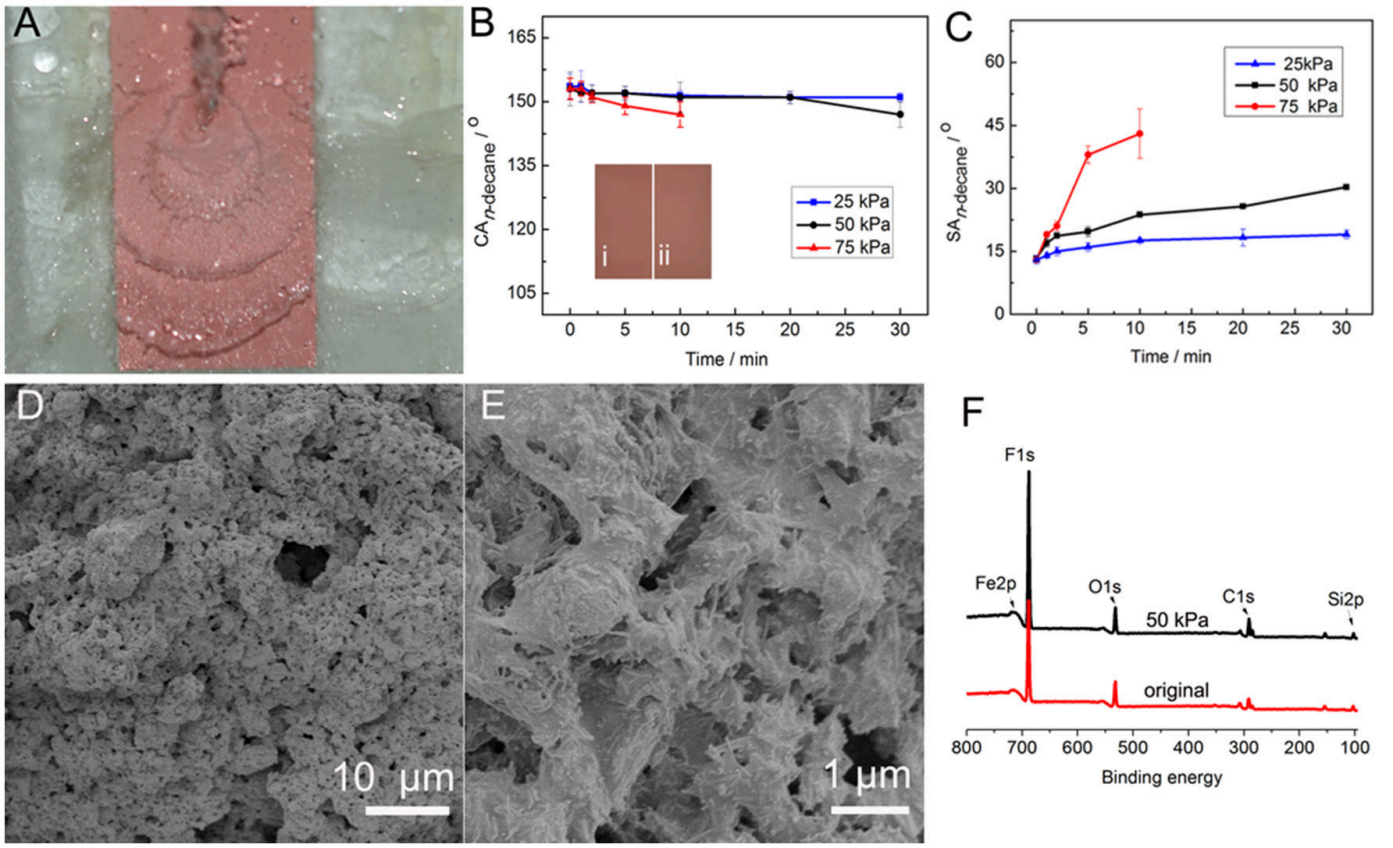
**(A)** Water jetting test at 50 kPa, and variation of **(B)** CA_n−decane_ and **(C)** SA_n−decane_ of the PAL/IOR@fluoroPOS coating with water jetting pressure and water jetting time. **(D,E)** SEM images and **(F)** XPS spectra of the coating after water jetting at 50 kPa for 30 min with the original coating for comparison. The insets in **(B)** are (I) the original coating and (II) the coating after water jetting at 50 kPa for 30 min. *C*_PAL/IOR_ = 14 g L^−1^, *t*_grinding_ = 20 min, *C*_PFDTES_ = 27.2 mM, *C*_TEOS_ = 4.5 mM.

Mechanical stability of the coating was tested by the water jetting test (Figure 7A). Water at certain pressure scoured the 45° tilted coating for a period of time, and then the superamphiphobicity was analyzed. The influences of water jetting pressure and water jetting time on the CA_n−decane_ and SA_n−decane_ are shown in Figures 7B,C. With increasing the water jetting time to 30 min at 25 kPa, the CA_n−decane_ did not show obvious change and the SA_n−decane_ slightly increased from 13 to 19°. The increase in the jetting pressure from 25 to 50 kPa had no influence on the CA_n−decane_ in the first 20 min, and then the CA_n−decane_ decreased to 147° in 30 min. However, the SA_n−decane_ at 50 kPa was obviously higher than that at 25 kPa, and increased to 30.3° after water jetting at 50 kPa for 30 min. This is because the SA is more sensitive to the changes of microstructure and chemical composition of super anti-wetting coatings than CA. (Li and Zhang, 2016) A higher water jetting pressure has more evident influence on the superamphiphobicity. For example, the SA_n−decane_ quickly increased to 43° after water jetting at 75 kPa for 10 min. The *n*-decane droplets became sticky on the coating with further increasing the jetting time at 75 kPa. Also, the mechanical stability of the coating was tested by the falling sand test (Supplementary Figure S6; Zhang and Seeger, 2011b). The sand particles 100–300 μm in diameter released from a height of 35 cm impacted the coating. The results indicate that the coating can withstand impact of 30 g of sand, and the n-decane drops still could roll down the coating.

After the water jetting test, the morphology and surface chemical composition of the coating were analyzed. After water jetting at 50 kPa for 30 min, no obvious difference between the original coating and the scoured coating can be seen according to their digital images (inset in Figure 7B). Also, there is no visible change in the micro-/nanostructure of the coating (Figures 7D,E). Moreover, the F 1s peak became stronger, and the F content is as high as 48.99 at.%, 2.51 at.% higher than the original coating (Figure 7F). This is because the bigger PAL/IOR@fluoroPOS aggregates with a higher PAL/IOR content on the surface of the coating have been washed away in the water jetting test. The SEM and the XPS results indicate high mechanical stability of the coating.

Chemical stability of the coating was investigated by immersing the coating in various corrosive solutions and organic solvents (Table 2). The coating showed distinctive resistance to all the liquids including strong acid, strong alkali, concentrated salt solution and organic solvents. After immersing in these liquids for 1 h, there were only slight changes in the CA_n−decane_ and SA_n−decane_. With increasing the time to 24 h, the coating was still superamphiphobic and the *n*-decane droplets could roll off the coating in spite of increase in the SA_n−decane_ (Supplementary Table S2). The XPS analysis indicates that immersion in these corrosive liquids for 24 h did not cause any obvious change in surface chemical composition of the coating (Supplementary Figure S5). The increase in the SA_n−decane_ is because of decrease of the surface roughness (Supplementary Figure S7). Interestingly, after UV irradiation for 24 h, slight improvement in superamphiphobicity was recorded. UV irradiation can increase temperature of the coating, which may improve the superamphiphobicity by promoting the condensation of residual silanols in the coating. Moreover, the coating retained its superamphiphobicity after placed in the lab environment for 8 months. There is no visible change in color of the coating after all these treatments (Supplementary Figure S8). All these results indicate high chemical stability of the coating.

**Table 2:** Chemical stability of the PAL/IOR@fluoroPOS coating.

	**CA_**n*−*decane_/°**	**SA_**n*−*decane_/°**
Original coating	153 ± 2.7	11.8 ± 2.8
1 M HCl_(aq)_, 1 h	148 ± 5.0	15.3 ± 0.6
1 M NaOH_(aq)_, 1 h	153 ± 2.1	15.0 ± 1.2
Saturated NaCl_(aq)_, 1 h	152 ± 3.0	14.7 ± 2.5
98% H_2_SO_4_, 1 h	150 ± 1.1	13.7 ± 2.0
Saturated NaOH_(aq)_, 1 h	150 ± 1.8	14.7 ± 2.4
Ethanol, 1 h	152 ± 2.1	14.0 ± 1.2
Toluene, 1 h	151 ± 0.5	14.7 ± 0.6
UV irradiation (200 W, 200–400 nm, 24 h, 10 cm)	155 ± 2.3	11.3 ± 1.2

*CA_n−decane_ and SA_n−decane_ of the PAL/IOR@fluoroPOS coating after treatment under different conditions. C_PAL/IOR_ = 14 g L^−1^, t_grinding_ = 20 min, C_PFDTES_ = 27.2 mM, C_TEOS_ = 4.5 mM*.

### Thermal stability

**Figure 8:**
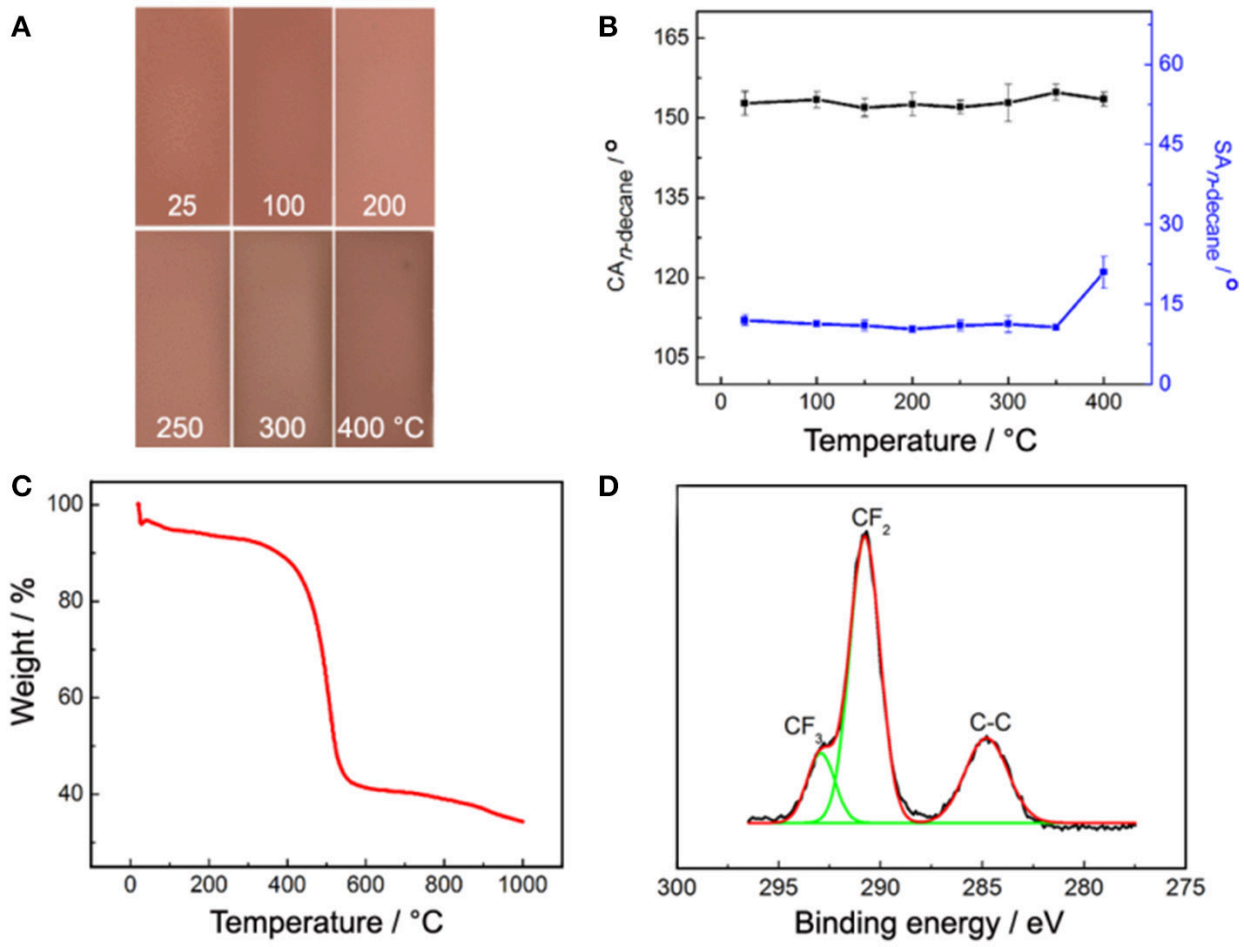
**(A)** Images and **(B)** CA_n−decane_ and SA_n−decane_ of the PAL/IOR@fluoroPOS coatings after kept at different temperature for 1 h, **(C)** TGA curve and **(D)** high-resolution C 1s XPS spectrum of the coating after kept at 400°C for 1 h. *C*_PAL/IOR_ = 14 g L^−1^, *t*_grinding_ = 20 min, *C*_PFDTES_ = 27.2 mM, *C*_TEOS_ = 4.5 mM.

Thermal stability of the PAL/IOR@fluoroPOS coating was investigated by oven test. The color of the coating remained unchanged at temperature up to 250°C for 1 h (Figure 8A). The coating faded a little at the edge when the temperature reached 400°C. In addition, the CA_n−decane_ remained 152°-154° up to 400°C. Meanwhile, the SA_n−decane_ slightly decreased from 12 to 10.7° with increasing the temperature to 350°C, and then increased to 21° at 400°C (Figure 8B). Annealing at proper temperature is helpful to improve superhydrophobicity of the silicone nanofilaments coatings by promoting the condensation of residual silanols. The decrease of the SA_n−decane_ of the PAL/IOR@fluoroPOS coating should be attributed to the same reason. However, a too high temperature caused partially oxidation of the perfluorodecyl groups of the coating, which resulted in decline of superamphiphobicity (Li et al., 2013). These results indicate much higher thermal stability of the coating than the reported colorful super anti-wetting coatings based on organic coloring species. The high thermal stability was also confirmed by the thermal gravimetric analysis (TGA, Figure 8C). The weight loss of the coating is low (12.4%) at temperature below 400°C, mainly owing to the loss of absorbed water. The weight loss is prominent in 400–530°C because of thermal oxidation and decomposition of the perfluorodecyl groups. After kept at 400°C for 1 h, the surface chemical composition of the coating was also analyzed. No change in the C 1s spectrum can be seen (Figures 3B, 8D). In addition, the F content on the surface of the coating is 46.35 at.%, only 1.3 at.% lower than that of the original coating.

### Colorful superamphiphobic coatings on different substrates

Besides glass slide, the PAL/IOR@fluoroPOS coatings are applicable onto different substrates including office paper, aluminum foil, wood plate and polyester textile via the same procedure (Supplementary Figure S9). All the coatings are superamphiphobic with small differences in the CA_n−decane_ and SA_n−decane_ (Supplementary Table S3). It should be noted that the CA_n−decane_ on the superamphiphobic polyester textile is lower than that on the other substrates. This is because the surfaces of textiles are macroscopically rough, and it is very difficult to detect the full drop profile for CA measurement (Zhang and Seeger, 2011a).

In addition, superamphiphobic coatings of different colors could be prepared according to the same procedure by using IOY, IOO, IOBR and IOBL instead of IOR. All these coatings have similar superamphiphobicity with droplets of soybean oil, *n*-dodecane and *n*-decane nearly spherical in shape on their surfaces (Supplementary Figure S10).

**Figure 9:**
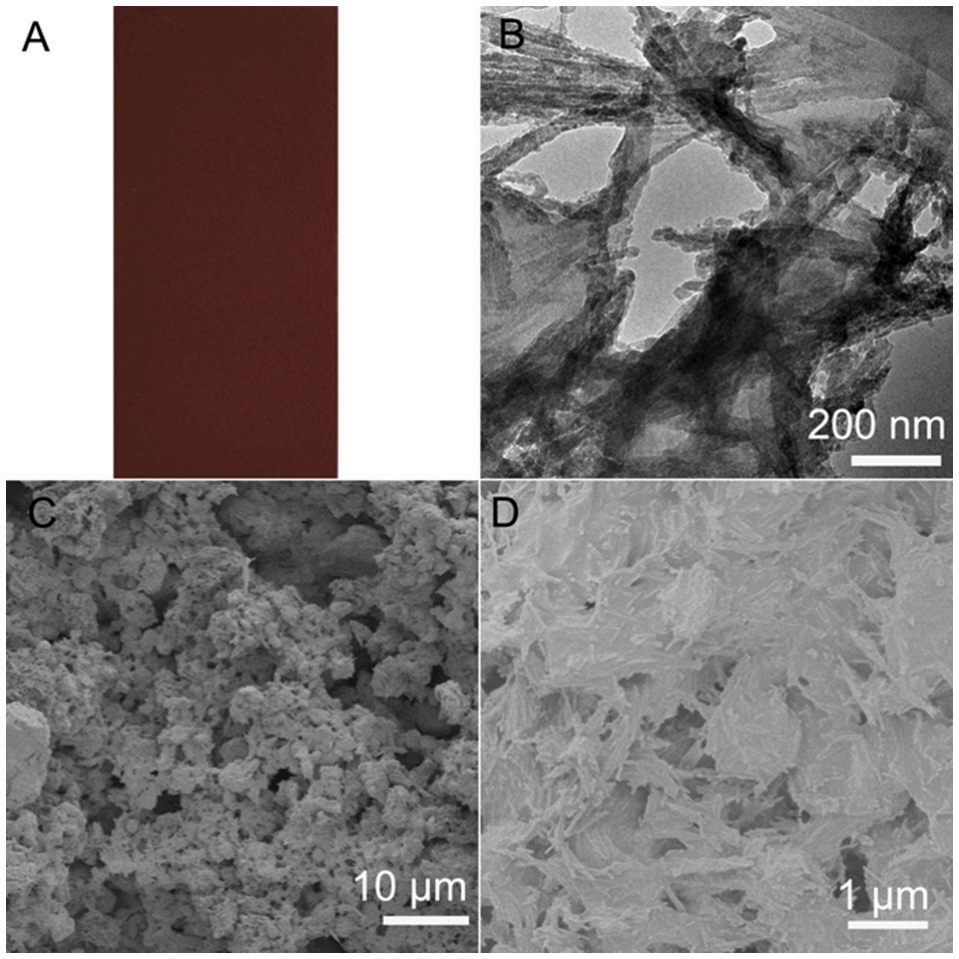
**(A**) Digital, **(B)** TEM **(C,D**) SEM images of the PAL/IOR@fluoroPOS coating from hydrothermal reaction of PAL with FeCl_3_·6H_2_O. *C*_PAL/IOR_ = 14 g L^−1^, *C*_PFDTES_ = 27.2 mM, *C*_TEOS_ = 4.5 mM.

Moreover, the PAL/IOR composite can also be prepared by *in situ* hydrothermal reaction of PAL with Fe(III) salts instead of solid-state grinding of PAL and IOR. Fe_2_O_3_ nanoparticles were formed on the PAL nanorods in the hydrothermal reaction (Tian et al., 2017). The color of the superamphiphobic coating is tunable by the dosage of Fe(III) in the hydrothermal reaction (Figure 9A). The CA_n−decane_ is 154° and the SA_n−decane_ is 10° on the coating, which means higher superamphiphobicity than the coating prepared by solid-state grinding. This is because hydrothermal reaction has no influence on the length of the PAL nanorods and the nanorods are kept very well (Figure 9B). In addition, different from the commercial IOR, the Fe_2_O_3_ nanoparticles are smaller and are uniformly anchored on the PAL nanorods. Thus, the nanoscale roughness of the coating is higher (Figures 9C,D), which is of benefit to the superamphiphobicity.

In fact, nanoparticles of metal oxides in different colors, such as blue CoAl_2_O_4_, yellow BiVO_4_ and white ZnO can be anchored on the PAL nanorods via specific reactions (Zhang et al., 2017). So, we believe that all these PAL-based nanocomposites can be used for preparing colorful superamphiphobic coatings via the same procedure. On the other hand, colorful superhydrophobic coatings could also be prepared via a similar procedure by using fluoro-free silanes, e.g., methyltrimethoxysilane and hexadecyltrimethoxysilane, instead of PFDTES (Reinen and Lindner, 1999; Zhang et al., 2016b).

Compared with the reported superamphiphobic coatings, our coatings showed higher superamphiphobicity and are among the coatings with the highest superamphiphobicity (Chu and Seeger, 2014). For example, the superamphiphobic coatings based on a mussel inspired dendritic polymer only has a CA_n−decane_ of 95° and a CA_n−hexadecane_ of 148° (Schlaich et al., 2016). None of these two liquids could roll off the coatings. Also, our method for the preparation of superamphiphobic coatings is more simple than most of the reported method, e.g., lithography and chemical etching (Liu and Kim, 2014; Cho et al., 2016). Moreover, the use of naturally abundant PAL as the building block could efficiently reduce the cost for preparation of superamphiphobic coatings. The cost of our coating is about CNY 70 per m^2^.

## Conclusion

In summary, a general method has been developed for the preparation of mechanically robust and thermally stable colorful superamphiphobic coatings. The superamphiphobicity is closely related to the surface microstructure and chemical composition of the coatings, which can be regulated by the concentrations of PAL/IOR, PFDTES, and TEOS and the solid-state grinding time. The colorful superamphiphobic coatings feature high contact angles and low sliding angles for water and various organic liquids with surface tension as low as 23.8 mN m^−1^. In comparison with the existing colorful super anti-wetting coatings, the coatings also feature high mechanical, chemical and thermal stability up to 400°C. Moreover, superamphiphobic coatings in different colors can be prepared via the same procedure, and are applicable onto various substrates. We believe that the findings here will shed light on the design of novel colorful super anti-wetting coatings. The colorful superamphiphobic coatings may find applications in many fields, such as anti-climbing of oils and restoration of cultural relics.

## Author contributions

NT performed the experimental design and data analysis, and wrote the manuscript; PZ performed experiments and contributed to the data analysis; JZ performed the experimental design and data analysis, and contributed to writing the manuscript.

### Conflict of interest statement

The authors declare that the research was conducted in the absence of any commercial or financial relationships that could be construed as a potential conflict of interest.

## References

[B1] Bellanger H. Darmanin T. Taffin de Givenchy E. Guittard F. (2014). Preparation of superoleophobic surfaces and related wetting theories. Chem. Rev. 114, 2694–2716. 10.1021/cr400169m 24405122

[B2] Bhushan B. Jung Y. C. (2011). Natural and biomimetic artificial surfaces for superhydrophobicity, self-cleaning, low adhesion, and drag reduction. Prog. Mater. Sci. 56, 1–108. 10.1016/j.pmatsci.2010.04.003

[B3] Cho H. Jeong J. Kim W. Choi D. Lee S. Hwang W. (2016). Conformable superoleophobic surfaces with multi-scale structures on polymer substrates. J. Mater. Chem. A 4, 8272–8282. 10.1039/C6TA02159J

[B4] Chu Z. Feng Y. Seeger S. (2015). Oil/water separation with selective superantiwetting/superwetting surface materials. *Angew. Chem. Int. Ed*. Engl. 54, 2328–2338. 10.1002/anie.201405785 25425089

[B5] Chu Z. Seeger S. (2014). Superamphiphobic surfaces. Chem. Soc. Rev. 43, 2784–2798. 10.1039/C3CS60415B 24480921

[B6] Deguchi S. Mukai S. Tsudome M. Horikoshi K. (2006). Facile generation of fullerene nanoparticles by hand-grinding. Adv. Mater. Weinheim. 18, 729–732. 10.1002/adma.200502487

[B7] Deng X. Mammen L. Butt H. J. Vollmer D. (2012). Candle soot as a template for a transparent robust superamphiphobic coating. Science 335, 67–70. 10.1126/science.1207115 22144464

[B8] Doménech A. Doménech-Carbó M. T. de Agredos Pascual M. L. V. (2007). Electrochemical monitoring of indigo preparation using Maya's ancient procedures. J. Solid State Electrochem. 11, 1335–1346. 10.1007/s10008-007-0296-2

[B9] Dong J. Wang Q. Zhang Y. Zhu Z. Xu X. Zhang J.. (2017). Colorful superamphiphobic coatings with low sliding angles and high durability based on natural nanorods. ACS App. Mater. Interfaces 9, 1941–1952. 10.1021/acsami.6b13539 28001033

[B10] Gao X. Jiang L. (2004). Biophysics: water-repellent legs of water striders. Nature 432, 36–36. 10.1038/432036a 15525973

[B11] Gu Z. Z. Uetsuka H. Takahashi K. Nakajima R. Onishi H. Fujishima A.. (2003). Structural color and the lotus effect. Angew. Chem. Int. Ed. 42, 894–897. 10.1002/anie.200390235 12596169

[B12] Koshkakaryan G. Klivansky L. M. Cao D. Snauko M. Teat S. J. Struppe J. O.. (2009). Alternative donor-acceptor stacks from crown ethers and naphthalene diimide derivatives: rapid, selective formation from solution and solid state grinding. J. Am. Chem. Soc. 131, 2078–2079. 10.1021/ja809088v 19161257

[B13] Li B. Zhang J. (2016). Durable and self-healing superamphiphobic coatings repellent even to hot liquids. Chem. Commun. 52, 2744–2747. 10.1039/C5CC09951J 26758697

[B14] Li B. Zhang J. Wu L. Wang A. (2013). Durable Superhydrophobic surfaces prepared by spray coating of polymerized organosilane/attapulgite nanocomposites. Chempluschem 78, 1503–1509. 10.1002/cplu.201300222 31986656

[B15] Li J. Hou Y. Liu Y. Hao C. Li M. Chaudhury M. K. (2016). Directional transport of high-temperature Janus droplets mediated by structural topography. Nat. Phys. 12, 606–612. 10.1038/nphys3643

[B16] Li J. Wu R. Jing Z. Yan L. Zha F. Lei Z. (2015). One-step spray-coating process for the fabrication of colorful superhydrophobic coatings with excellent corrosion resistance. Langmuir 31, 10702–10707. 10.1021/acs.langmuir.5b02734 26365307

[B17] Liu K. Yao X. Jiang L. (2010). Recent developments in bio-inspired special wettability. Chem. Soc. Rev. 39, 3240–3255. 10.1039/b917112f 20589267

[B18] Liu T. L. Kim C. J. (2014). Turning a surface superrepellent even to completely wetting liquids. Science 346, 1096–1100. 10.1126/science.1254787 25430765

[B19] Liu Y. Liu J. Li S. Liu J. Han Z. Ren L. (2013). Biomimetic superhydrophobic surface of high adhesion fabricated with micronano binary structure on aluminum alloy. ACS Appl. Mater. Interfaces 5, 8907–8914. 10.1021/am4014715 24016423

[B20] Liu Y. Wang W. Wang A. (2012). Effect of dry grinding on the microstructure of palygorskite and adsorption efficiency for methylene blue. Powder Technol. 225, 124–129. 10.1016/j.powtec.2012.03.049

[B21] Lu Y. Song J. Liu X. Xu W. Xing Y. Wei Z. (2012). Preparation of superoleophobic and superhydrophobic titanium surfaces via an environmentally friendly electrochemical etching method. ACS Sustain. Chem. Eng. 1, 102–109. 10.1021/sc3000527

[B22] Neinhuis C. Barthlott W. (1997). Characterization and distribution of water-repellent, self-cleaning plant surfaces. Ann. Bot. 79, 667–677. 10.1006/anbo.1997.0400

[B23] Ogihara H. Okagaki J. Saji T. (2011). Facile fabrication of colored superhydrophobic coatings by spraying a pigment nanoparticle suspension. Langmuir 27, 9069–9072. 10.1021/la200898z 21718077

[B24] Papadopoulos P. Vollmer D. Butt H.-J. (2016). Long-term repellency of liquids by superoleophobic surfaces. Phys. Rev. Lett. 117:046102. 10.1103/PhysRevLett.117.046102 27494484

[B25] Qu M. Ma X. He J. Feng J. Liu S. Yao Y.. (2016). Facile selective and diverse fabrication of superhydrophobic, superoleophobic-superhydrophilic and superamphiphobic materials from kaolin. ACS Appl. Mater. Interface 9, 1011–1020. 10.1021/acsami.6b10964 27959496

[B26] Reinen D. Lindner G.-G. (1999). The nature of the chalcogen colour centres in ultramarine-type solids. Chem. Soc. Rev. 28, 75–84. 10.1039/a704920j

[B27] Sato O. Kubo S. Gu Z.-Z. (2008). Structural color films with lotus effects, superhydrophilicity, and tunable stop-bands. Account Chem. Res. 42, 1–10. 10.1021/ar700197v 18837520

[B28] Schlaich C. Cuellar Camacho L. Yu L. Achazi K. Wei Q. Haag R. (2016). Surface-independent hierarchical coatings with superamphiphobic properties. ACS Appl. Mater. Interfaces 8, 29117–29127. 10.1021/acsami.6b08487 27714994

[B29] Soler R. Salabert J. Sebastián R. M. Vallribera A. Roma N. Ricart S.. (2011). Highly hydrophobic polyfluorinated azo dyes grafted on surfaces. Chem. Commun. 47, 2889–2891. 10.1039/c0cc04695g 21243125

[B30] Steele A. Bayer I. Loth E. (2008). Inherently superoleophobic nanocomposite coatings by spray atomization. Nano Lett. 9, 501–505. 10.1021/nl8037272 19099463

[B31] Tian G. Wang W. Mu B. Wang Q. Wang A. (2017). Cost-efficient, vivid and stable red hybrid pigments derived from naturally available sepiolite and halloysite. Ceram. Int. 43, 1862–1869. 10.1016/j.ceramint.2016.10.145

[B32] Tian X. Verho T. Ras R. H. (2016). Moving superhydrophobic surfaces toward real-world applications. Science 352, 142–143. 10.1126/science.aaf2073 27124437

[B33] Tuteja A. Choi W. Ma M. Mabry J. M. Mazzella S. A. Rutledge G. C.. (2007). Designing superoleophobic surfaces. Science 318, 1618–1622. 10.1126/science.1148326 18063796

[B34] Wang Y. M. Wu Z. Y. Wang H. J. Zhu J. H. (2006). Fabrication of metal oxides occluded in ordered mesoporous hosts via a solid-state grinding route: the influence of host-guest interactions. Adv. Funct. Mater. 16, 2374–2386. 10.1002/adfm.200500613

[B35] Wong W. S. Liu G. Nasiri N. Hao C. Wang Z. Tricoli A. (2017). Omnidirectional self-assembly of transparent superoleophobic nanotextures. ACS Nano 11, 587–596. 10.1021/acsnano.6b06715 28027438

[B36] Zhang A. Mu B. Luo Z. Wang A. (2017). Bright blue halloysite/CoAl_2_O_4_ hybrid pigments: preparation, characterization and application in water-based painting. Dyes Pigm. 139, 473–481. 10.1016/j.dyepig.2016.12.055

[B37] Zhang J. Li B. (2013). Universal dispersion of single-walled carbon nanotubes in the liquid phase inspired by Maya Blue. J. Mater. Chem. A 1, 10626–10630. 10.1039/c3ta12284k

[B38] Zhang J. Seeger S. (2011a). Polyester materials with superwetting silicone nanofilaments for oil/water separation and selective oil absorption. Adv. Funct. Mater. 21, 4699–4704. 10.1002/adfm.201101090

[B39] Zhang J. Seeger S. (2011b). Superoleophobic coatings with ultralow sliding angles based on silicone nanofilaments. Angew. Chem. Int. Ed. 50, 6652–6656. 10.1002/anie.201101008 21648031

[B40] Zhang Y. Dong J. Sun H. Yu B. Zhu Z. Zhang J.. (2016a). Solvatochromic coatings with self-cleaning property from palygorskite@ polysiloxane/crystal violet lactone. ACS Appl. Mater. Interfaces 8, 27346–27352. 10.1021/acsami.6b09252 27657669

[B41] Zhang Y. Fan L. Chen H. Zhang J. Zhang Y. Wang A. (2015). Learning from ancient Maya: preparation of stable palygorskite/methylene blue@SiO_2_ Maya Blue-like pigment. Micropor. Mesopor. Mater. 211, 124–133. 10.1016/j.micromeso.2015.03.002

[B42] Zhang Y. Zhang J. Wang A. (2016b). From Maya blue to biomimetic pigments: durable biomimetic pigments with self-cleaning property. J. Mater. Chem. A 4, 901–907. 10.1039/C5TA09300G

[B43] Zimmermann J. Rabe M. Artus G. R. Seeger S. (2008). Patterned superfunctional surfaces based on a silicone nanofilament coating. Soft Matt. 4, 450–452. 10.1039/b717734h 32907202

