# Supplementary material for: Mechanically Robust and Thermally Stable Colorful Superamphiphobic Coatings
Source: Front Chem. 2018 Apr 30;6:144. doi: 10.3389/fchem.2018.00144 (PMC5936788; doi:10.3389/fchem.2018.00144)
Supplement: Supplementary file 1 [file Data_Sheet_1.PDF]

Supplementary Material

Mechanically Robust and Thermally Stable Colorful Superamphiphobic Coatings

Ning Tian,[1,2] Penglin Zhang,[2] and Junping Zhang[1*]

*** Correspondence:** Junping Zhang jpzhang@licp.cas.cn

1 Supplementary Figures and Tables

1.1 Supplementary Figures

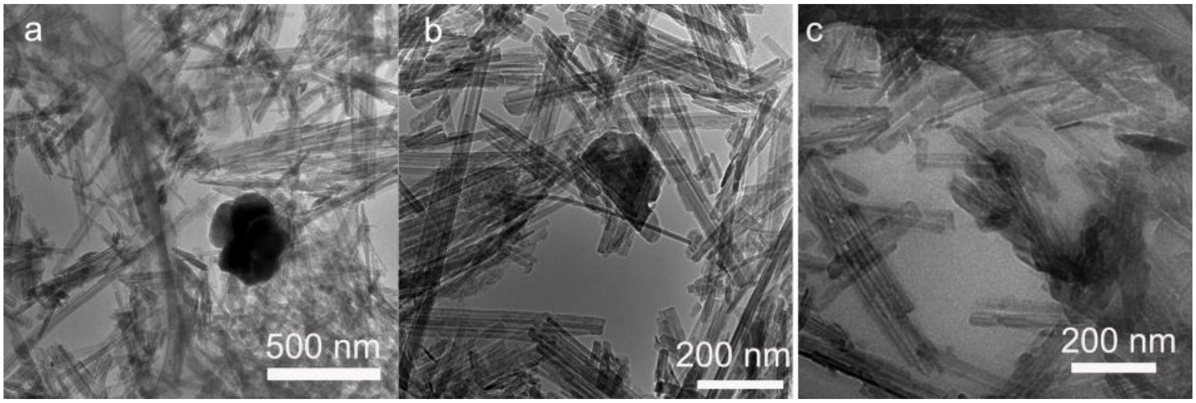

Supplementary Figure S1. TEM images of (a, b）PAL/IOR and (c) PAL/IOR@fluoroPOS.

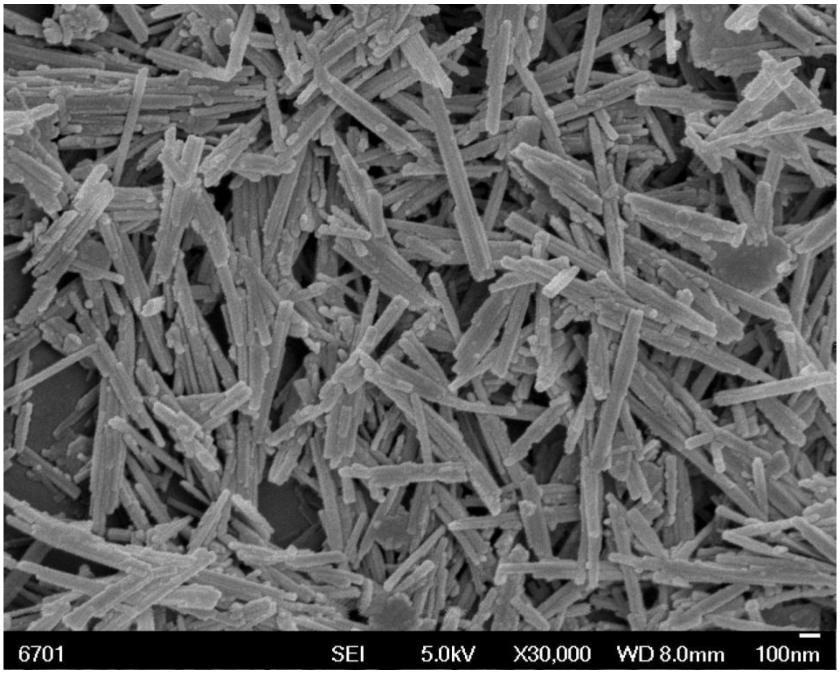

Supplementary Figure S2. SEM images of PAL.

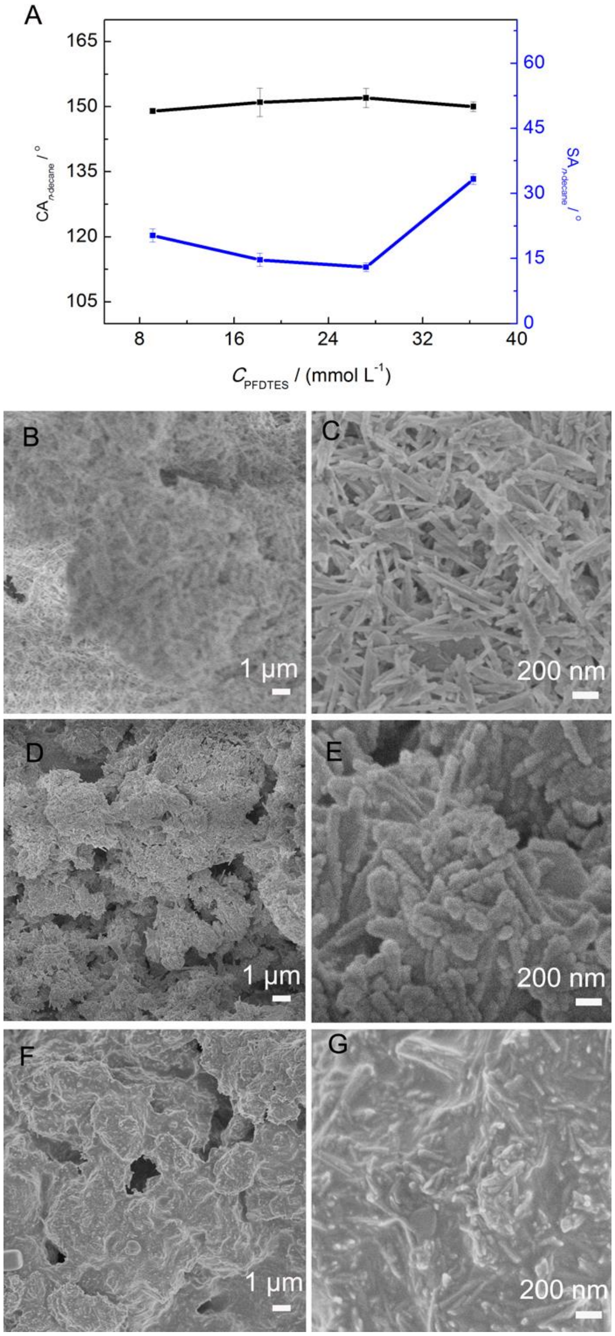

Supplementary Figure S3 (a) Variation of $CA_{n\text{-decane}}$ and $SA_{n\text{-decane}}$ of the PAL/IOR@fluoroPOS coatings with C_{PFDTES}. SEM images of the PAL/IOR@fluoroPOS coatings with a C_{PFDTES} of (b-c) 9.1 mM, (d-e) 27.2 mM and (f-g) 36.3 mM. $C_{PAL/IOR}$ = 14 g L^{-1}, $t_{grinding}$ = 20 min, C_{TEOS} = 4.5 mM.

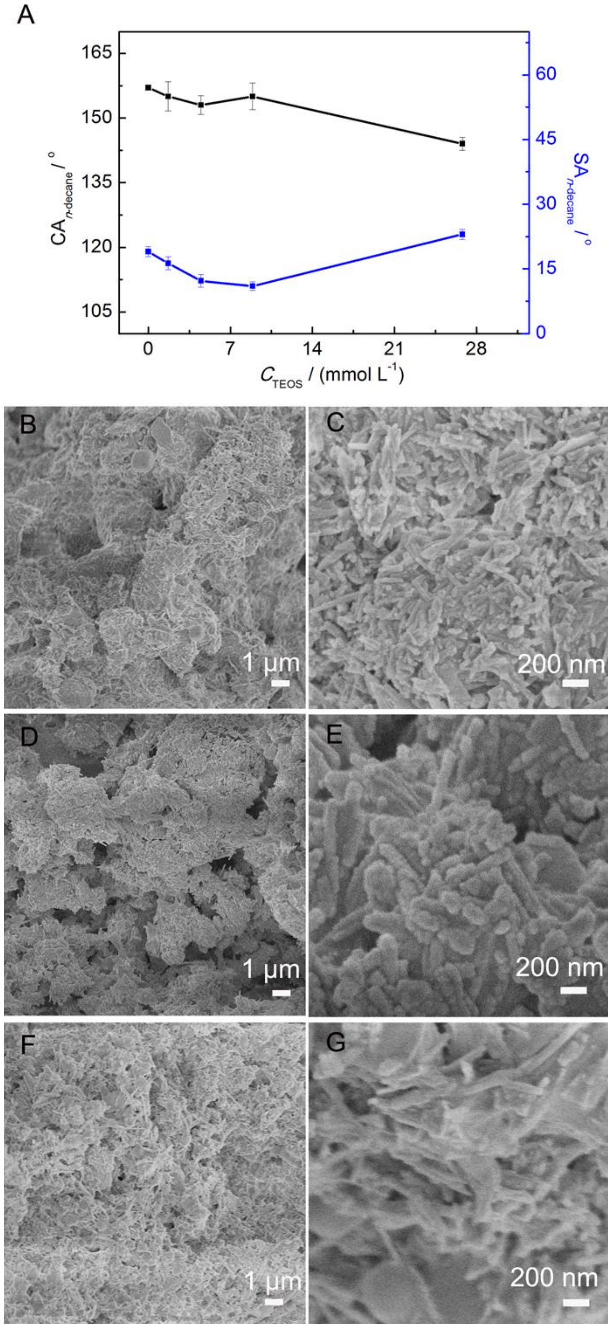

Supplementary Figure S4. (a) Variation of $CA_{n\text{-decane}}$ and $SA_{n\text{-decane}}$ of the PAL/IOR@fluoroPOS coatings with C_{TEOS}. SEM images of the PAL/IOR@fluoroPOS coatings with a C_{TEOS} of (b-c) 0 mM, (d-e) 4.5 mM and (f-g) 8.9 mM. $C_{PAL/IOR}$ = 14 g L^{-1}, $t_{grinding}$ = 20 min, C_{PFDTES} = 27.2 mM.

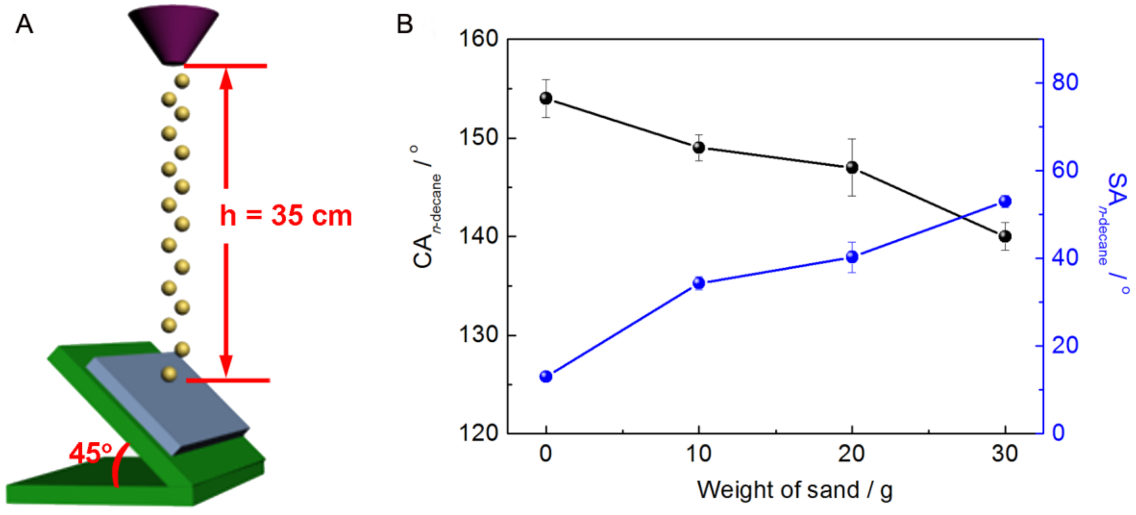

Supplementary Figure S5. (a) Schematic illustration of the falling sand test. (b) Variation of CA$_{n\text{-decane}}$ and SA$_{n\text{-decane}}$ of the PAL/IOR@fluoroPOS coating with weight of sand in the falling sand test.

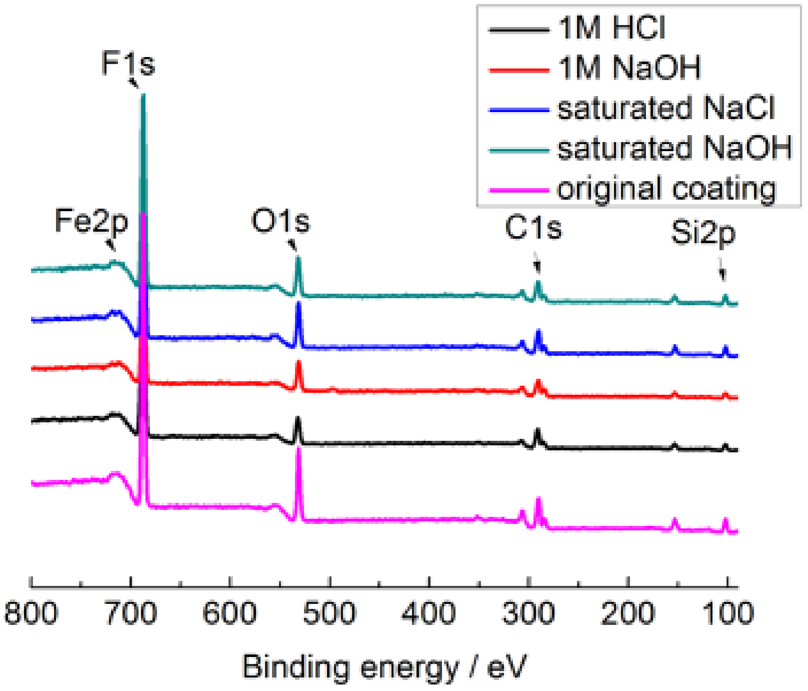

Supplementary Figure S6. (a) XPS spectra of the PAL/IOR@fluoroPOS coatings after immersed in various liquids for 24 h with the original coating for comparison. $C_{\mathrm{PAL/IOR}}$ = 14 g L^{-1}, t_{grinding} = 20 min, C_{PFDTES} = 27.2 mM, C_{TEOS} = 4.5 mM.

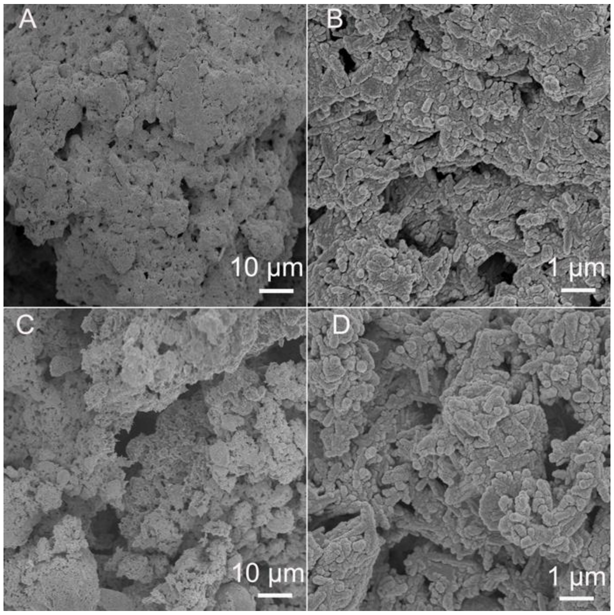

Supplementary Figure S7. SEM images of PAL/IOR@fluoroPOS coatings after immersion in (a-b) 1 M HCl and (c-d) 1M NaOH for 24 h. $C_{\mathrm{PAL/IOR}}$ = 14 g L^{-1}, t_{grinding} = 20 min, C_{PFDTES} = 27.2 mM, C_{TEOS} = 4.5 mM.

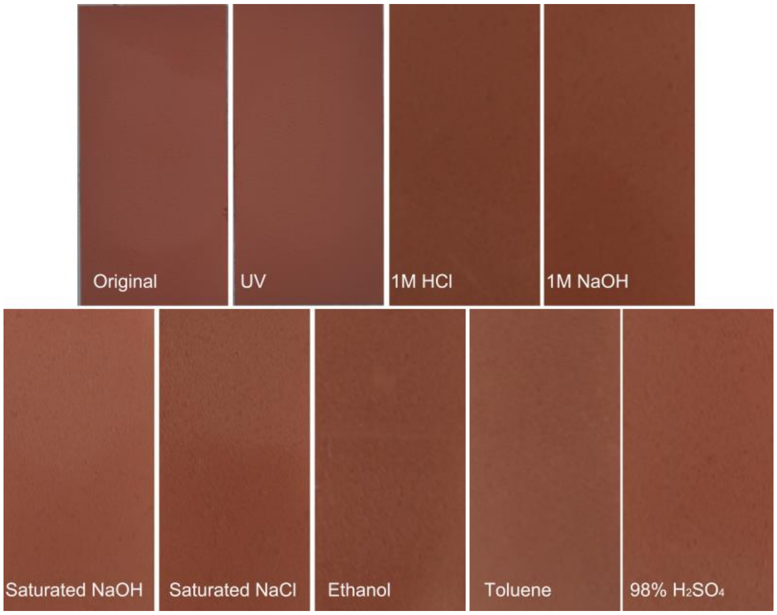

Supplementary Figure S8. Digital images of the PAL/IOR@fluoroPOS coatings after immersed in different liquids or exposed to UV irradiation for 24 h. $C_{\mathrm{PAL/IOR}}$ = 14 g L^{-1}, t_{grinding} = 20 min, C_{PFDTES} = 27.2 mM, C_{TEOS} = 4.5 mM.

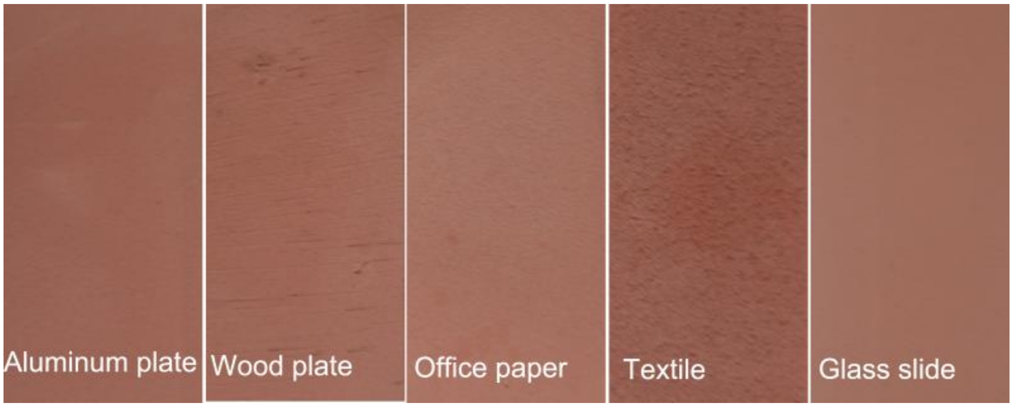

Supplementary Figure S9. PAL/IOR@fluoroPOS coatings on different substrates. $C_{PAL/IOR}$ = 14 g L^{-1}, $t_{grinding}$ = 20 min, C_{PFDTES} = 27.2 mM, C_{TEOS} = 4.5 mM.

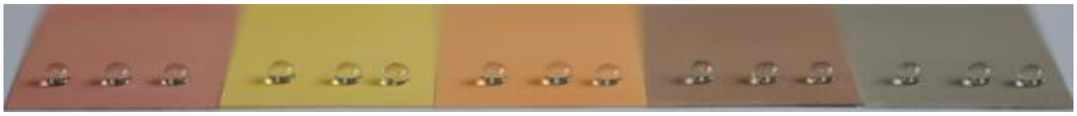

Supplementary Figure S10. Soybean oil, *n*-dodecane and *n*-decane droplets (left to right) on the superamphiphobic coatings based on (a) IOR, (b) IOY, (c) IOO, (d) IOBR and (e) IOBL. $C_{PAL/iron\ oxides}$ = 14 g L^{-1}, $t_{grinding}$ = 20 min, C_{PFDTES} = 27.2 mM, C_{TEOS} = 4.5 mM.

1.2 Supplementary Tables

Supplementary Table S1. CAs and SAs of the frequently used oils in our daily life on the PAL/IOR@fluoro POS coating at 20 ℃. $C_{PAL/IOR}$ = 14 g L^{-1}, $t_{grinding}$ = 20 min, C_{PFDTES} = 27.2 mM, C_{TEOS} = 4.5 mM.

Liquids	CAs / °	SAs / °
Rapeseed oil	160 ±2.5	7.2 ±1.4
Diesel	163 ±2.3	8.5 ±1.2
Vacuum pump oil	158 ±2.6	9.7 ±0.58
Soybean oil	160 ±2.5	15.0 ±1.2

Supplementary Table S2. $CA_{n\text{-decane}}$ and $SA_{n\text{-decane}}$ of the PAL/IOR@fluoroPOS after treatment under different conditions for 24 h. $C_{PAL/IOR}$ = 14 g L^{-1}, $t_{grinding}$ = 20 min, C_{PFDTES} = 27.2 mM, C_{TEOS} = 4.5 mM.

	$CA_{n\text{-decane}}$ / °	$SA_{n\text{-decane}}$ / °
Original coating	153 ±2.7	11.8 ±2.8
1 M $HCl_{(aq)}$, 24 h	145 ±2.5	32.7 ±2.1
1 M $NaOH_{(aq)}$, 24 h	145 ±2.7	32.0 ±2.1
Saturated $NaCl_{(aq)}$, 24 h	146 ±4.0	19.0 ±0.6
98 % H_2SO_4, 24 h	150 ±3.0	20.0 ±1.5
Saturated $NaOH_{(aq)}$, 24 h	148 ±2.0	29.7 ±0.6
Ethanol, 24 h	147 ±1.9	28.0 ±4.2
Toluene, 24 h	149 ±1.2	19.7 ±0.6

Supplementary Table S3. $CA_{n\text{-decane}}$ and $SA_{n\text{-decane}}$ of the PAL/IOR@fluoroPOS coatings on different substrates. $C_{PAL/IOR}$ = 14 g L^{-1}, $t_{grinding}$ = 20 min, C_{PFDTES} = 27.2 mM, C_{TEOS} = 4.5 mM.

Substrates	$CA_{n\text{-decane}}$ / °	$SA_{n\text{-decane}}$ / °
Glass slide	153 ±2.7	11.8 ±2.8
Office paper	150 ±3.1	13.3 ±1.2
Aluminum foil	152 ±2.3	17.5 ±1.5
Wood plate	154 ±4.0	19.0 ±0.6

Polyester textile	146 ± 3.2	16.5 ± 1.5
